# Progress in Medicine

**Published:** 1898-12-17

**Authors:** 


### Progress in Medicine.

### DIPHTHERIA.

(*Continued from page 179.*)

**Vitality of the Diphtheria Bacillus.**—Macgregor[16] reports a case of a boy, aged eight years, in which the Klebs-Löffler bacilli were present in a virulent condition nearly six months after the attack of diphtheria. He cites other instances of a similar kind. Thus Schäfer[17] records a case where bacilli found in the throat, examined seven and a half months after the attack, proved virulent by inoculation of animals. Tobeisen[18] found diphtheria bacilli in the throats of 24 out of 46 patients at the time of their discharge from the hospital, and in 19 out of the 24 cases the bacilli proved virulent. Bigg states that "in 245 cases out of 405 cases of true diphtheria the diphtheria bacilli disappeared within three days after the complete separation of the false membrane; in 160 cases they persisted for a longer time, namely, in 103 cases for seven days, in 34 cases for 12 days, in 16 cases for 15 days, in four for three weeks, and in three for five weeks, after the time when the exudation had completely disappeared from the upper passages." The author further cited the statement of Hewlett and Knight[19] that they were sometimes able to convert the non-virulent pseudo-diphtheria bacillus into a virulent Klebs-Löffler bacillus; and that by careful treating it has apparently been possible to convert a typical Klebs-Löffler into a typical non-virulent pseudo-bacillus.

To get rid of the bacilli A. C. Abbott,[20] director of the Bacteriogical Laboratory, Philadelphia, recommends thorough application once daily, to the parts that have been affected, of a solution of 60 grains of silver nitrate to the ounce of water. Two applications are usually sufficient, but occasionally a third is necessary. Weaker solutions were found to be more painful. It should not be used until the membrane has disappeared, as otherwise the coagulum formed might protect organisms below it.

**Diphtheria Bacilli in the Urine.**—F. Smith[21] has found bacilli virulent in the urine of guinea-pigs passing hæmorrhagic urine after inoculation. The facts show that in guinea-pigs, with the bacilli in the blood stream, some of them are eliminated living in the urine. The results suggest that, at least in hæmorrhagic diphtheria in human beings, bacilli will be found in the urine, and that in all cases in which the bacillus escapes into the blood stream it will be present in the urine. Those hygienists who have so strenuously maintained that there is a connection between general insanitation, middens, &c., and diphtheria will have a new fact to strengthen their argument.

**Influence of Season and of School Attendance on Mortality.**—F. A. Dixey,[22] by an extensive analysis of the mortality rates from diphtheria in London during the preceding ten years, has shown that the mortality rate rises almost uniformly in the autumn, ranging from 40 per cent. to 54 4 per cent. during months from the middle of September to the middle of February, and falling to between 39 per cent. and 33 per cent. during the rest of the year. The influence on the spread of diphtheria of infection contracted at school seems evident when several years are taken in succession; for in eight years from 1891 to 1898 the bottom of the first or January depression was reached four times in the third week, twice in the fourth; while on two more occasions it was deferred until the sixth. In the seven years from 1891 to 1897 the second or August depression reached its lowest point four times in the thirty-fifth week, twice in the thirty-sixth, and once in the thirty-fourth. Looking at the facts as a whole, Dixey does not see any way of escaping the inference that there is a factor at work, distinct from ordinary seasonal conditions, which is sufficiently powerful to induce a fairly regular disturbance of the course of fatal diphtheria at two definite times of the year, these times corresponding with the usual holiday periods of schools. The author gives further references to the writings of other observers on this point.

Mr. Shirley Murphey, in his report to the Public Health Committee shows that the result of his investigations lead to the same conclusions as regards school attendance, and he further shows that this influence is not confined to London, but holds good in a number of other towns in this country and at Berlin.

**Post-Diphtherial Paralysis.**—A discussion on this question was opened by Sims Woodhead[23] at the Edinburgh Annual Meeting of the British Medical Association. The importance of this subject may be gathered from the fact that during the year 1896 there were examined at the laboratories of the Royal College of Physicians and Surgeons of London, 7,832 cases that had been certified "diphtheria," and of these cases 5,068 had diphtheria bacilli in the throat and 1,362 suffered from paralysis of a more or less marked kind. Of these cases, 1,096 had been treated with anti-toxin, and there were 273 deaths amongst them; 266 received no anti-toxin (probably they were therefore mostly mild cases), and there were 49 deaths. In 1,764 of the cases, in which no diphtheria bacilli were found, there were 177 cases of paralysis with 59 deaths; 89 of these cases were treated with anti-toxin —31 deaths. There were, moreover, 88 not treated with anti-toxin, 28 of these succumbing. The author cites A.

Miller's[24] account of a number of cases of paralysis observed by him at the South-Eastern Hospital during the years 1896 and 1897, in which the onset was primarily (1) in the muscles of the palate; (2) in the oculo-motor muscles; (3) in the muscles of other parts; and (4) in cases of paralytic heart failure. He collects 494 cases, of which 185 were primary paralysis of the palate, 197 strabismus, 10 paralysis of other muscles, 102 cases of cardiac paralysis, 91 of which died and 11 recovered. The bulk of the palatal paralysis occurred between the fifth and the fifteenth day, none before the fourth. Of the oculo-motor paralyses the bulk occurred between the fourth and seventeenth days, none before the fourth; of the primary paralyses of other parts, half of them occurred between the tenth and fourteenth days, and none before the earlier day. A case of primary paralysis of the palate occurred as late as the sixty-fifth day; a primary oculo-motor paralyses the ninety-first day, and primary paralysis of other parts on the fifty-first day. The bulk of the cardiac paralysis occurred between the fifth and the tenth days; a few cases occurred even as early as the second day, whilst this condition occurred in a severe form (that is, ending fatally) in two cases on the fifty-fourth day, and in one case which recovered on the fifty-ninth day. Sims Woodhead went on to say that it is evident, then, that in the human subject these paralyses occur at a comparatively early date, although in numerous cases they come on at very much later stages; and one cannot help thinking that we have evidence of the primary affection of the nerve cells or of a direct action of the poison on the muscular tissue in the fact that cardiac paralysis occurred relatively at so much earlier a period than the other forms of paralysis. The affection of the cells of the vaso-motor centres, and perhaps also of the ganglia of the heart being much more important in the regulation of the heart muscle, which is constantly in action, than in the case of the other muscles, which in most cases are practically at rest, and in which therefore the demands on both the motor cells and the muscular tissue itself are comparatively slight. Woodhead then dealt with the morbid histology, and, without going into the theory of the causation of paralysis, he referred to the segmental nerve degeneration described by Meyer, Sidney Martin, Dejerine, and others. Comparatively recently, attention has been drawn to the fact that certain slight and perhaps temporary changes may be observed during the first twenty-four hours in which toxin may be acting on the nerve cells. It is maintained that the cells so affected either atrophy or recover at an early date, but that the nerves for which these cells act as trophic centres undergo degenerative changes, perhaps of a Wallerian type, and that only then do we meet with the characteristic diphtheritic paralysis.

F. W. Mott, in the course of the discussion, remarked that in five cases of diphtherial paralysis he had examined he had found generally fatty degeneration, early and late, of the muscles, and sometimes Wallerian degeneration of the nerves. He had observed extreme early fatty degeneration of the heart, and yet no degeneration of the vagus nerve. He was of opinion that probably the poison acted on the whole neuron, especially upon the terminal arborisations of the dendron and the end plates. The early cardiac failure, he thought, could be best explained by the effect of stress. The heart muscle and respiratory muscles could not rest, but even had to work harder. They would, therefore, more readily succumb to the action of the poison.

Baginsky stated that his assistant, Dr. Katz, had examined the brain, spinal cord, and peripheral nerves, and found advanced degenerative changes in all parts of the nervous system, both nerve cells and nerve fibres. Extreme degeneration of the muscle fibres of the heart and diaphragm was also found. In regard to post-diphtherial hemiplegia, he thought that cerebral embolism was the most usual cause. He had found this condition present in three such cases examined, and thought that a distinction should be made between them and cases of real diphtherial paralysis. In his opinion, early anti-toxin treatment was of the greatest value in diminishing the number of cases of paralysis and diphtheria.

Dr. E. W. Goodall (London) said that since the introduction of the anti-toxin treatment the incidence of paralysis following diphtheria had certainly increased. The reason of this we believe to be that patients now recovered, or, at any rate, lived long enough to show symptoms of paralysis, who without anti-toxin would have died at an earlier period. If the serum treatment were commenced early enough the number of cases of paralysis would be lower instead of higher than before. This was well shown in the post-scarlatinal diphtheria cases in the Metropolitan Asylums Board's hospitals during 1896. These cases were all under skilled observation from the very commencement, and the serum treatment was begun early. Among them the number of paralysis cases was considerably less among the diphtheria cases admitted from outside during the same period, and there was only one case fatal from paralysis.

F. E. Batten[25] has examined five cases of diphtherial paralysis by the March's method, examining the cranial nerves, the spinal cord, the anterior and posterior nerve roots, the posterior root ganglia, the vagi, the phrenics, and some of the nerves to the upper and lower extremities. He also examined the spinal cord and the posterior root ganglia by Nissl's method, and the nerves by Pal's method. All the cases gave a positive result except one, which was a case of hemiplegia due to softening of the lenticular nucleus. The result of the examination is as follows: He finds degeneration of a parenchymatous nature in various cranial nerves in the anterior and posterior nerve roots, and in the nerve fibres as they pass through the white matter to the grey matter of the spinal cord, in the vagus, phrenic and peripheral nerves, and also on both sides of the posterior root ganglia. By Nissl's method he examined the spinal cord and the posterior root ganglia, and the cells in both places were found perfectly normal. By Pal's method no change could be demonstrated. The paper gives a short digest of the work of various authors on the same subject; he refers to the work of Martin, who has shown that the finer nerve branches of the peripheral nerves are primarily affected, and to the more recent work of Mouravjeff, which tends to show that the primary alteration occurs in the cells of the anterior horn (demonstrated by Nissl's method), the parenchymatous change in the nerves being a secondary and later change; the same had also been shown by Crocq. The affection of the posterior roots was first shown by Meyers in 1881, and he found that degeneration existed on both sides of the posterior root ganglia, and this has again been more recently noted by Crocq, Priez, Mouravjeff, and others. Bikeles described the occurrence of fat granules in the posterior roots at their entrance into the cord; the condition described is probably a normal condition commonly present in children, and to a lesser degree in adults. In conclusion, the author regards the dominant lesion in diphtheritic paralysis as a parenchymatous degeneration of the myelin sheath affecting both sensory and motor elements.

Brannan[26] reports one of those rare cases of post-diphtheritic paralysis, cerebral hemiplegia. The patient recovered. The author states that there are 35 other cases in all recorded in medical literature, while his own was the only case known among the thousands of patients treated at the Willard Park Hospital during the past few years. In the 35 reported cases the lesion was thought to be hæmorrhage in seven cases, and embolism in 14; in the other 14 the diagnosis was uncertain. Six cases have come to autopsy. In one of these a hæmorrhage was found in the internal portion of the lenticular nucleus, with destruction of the neighbouring part of the internal capsule. In the other five cases there was embolism of the Sylvian artery. Whether the vascular lesion be hæmorrhage, thrombosis, or embolism, it is probably situated in the internal capsule in the great majority of cases. Post-diphtheritic cerebral hemiplegia is most common in children from eight to fifteen years of age. The prognosis is grave, as in similar lesions in adults. Hunter[27] also records a case of hemiplegia due to diphtheria, which was associated with the more usual peripheral diphtherial paralysis.

[16] Lancet, Mar. 12, 1898. [17] Brit. Med. Jour., 1895, vol. I, p. 65. [18] Centralbl. f. Bakt., 1892, vol. xii., p. 587. [19] Transactions of the Institute of Prev. Med., 1897. [20] Philadelphia Med. Jour., Aug. 27, 1898; Treatment, Nov. 10, 1898. [21] Lancet, Nov. 19 1898. [22] Brit Med. Jour., Sept. 3, 1898. [23] Brit. Med. Jour., Sept. 3, 1898. [24] Med. Suppt. to the Report for the year 1897 of the Statistical Committee of the Metropolitan Asylums Board 1898, p. 135. [25] Brit. Med. Jour., Nov. 19, 1898. [26] New York Med. Rec., July 30, 1898. [27] Glasgow Med. Jour., Nov., 1898.

